# Gangliosides in molecular interactions and cell regulation

**DOI:** 10.1016/j.jbc.2026.111184

**Published:** 2026-01-23

**Authors:** Kristina Mlinac-Jerkovic, Marija Heffer, Ronald L. Schnaar

**Affiliations:** 1 Croatian Institute for Brain Research, University of Zagreb, Zagreb, Croatia; 2 Department of Chemistry and Biochemistry School of Medicine, University of Zagreb, Zagreb, Croatia; 3 Department of Medical Biology and Genetics, Faculty of Medicine, University of Osijek, Osijek, Croatia; 4 Department of Physiology, Pharmacology & Therapeutics, Johns Hopkins University School of Medicine, Baltimore, Maryland, USA; 5 Department of Neuroscience, Johns Hopkins University School of Medicine, Baltimore, Maryland, USA

**Keywords:** bacterial toxin, cancer, glycosphingolipid, lipid raft, neurodegeneration, sialic acid

## Abstract

Gangliosides are sialoglycolipids expressed by all vertebrate cells. They are found predominantly on the outer leaflet of the plasma membrane but also on select intracellular membranes. As sphingolipids they dynamically associate into lipid rafts where they modulate the activity of receptors and ion channels and serve as ligands for sialoglycan binding proteins on other cells or in the extracellular milieu. Gangliosides support nervous system stability, regulate neurotransmitter and ion channel expression and activity, regulate receptor protein kinases, are responsible for select binding of toxins and pathogens, and have other molecular/cellular regulatory functions. Rare subjects with congenital disorders of ganglioside biosynthesis suffer severe and broad multi-system deficits. Changes in ganglioside expression are characteristic of cancer and neurodegenerative diseases, leading to targeting or use of gangliosides therapeutically. This review presents properties of gangliosides, mechanisms and examples of their physiological and pathological functions, and examples of their roles in human diseases.

Gangliosides are regulatory molecules expressed by all vertebrate cells and tissues. As glycolipids (more precisely glycosphingolipids) they reside in membranes – primarily the plasma membrane – with their lipid tails deeply embedded in the bilayer and their sialoglycans extending out from the cell surface (1). They contribute to the glycocalyx, the ubiquitous glycan coat on cells that constitutes each cell’s molecular face to the extracellular environment. Gangliosides have long been known as recognition molecules, first for pathogens and toxins and later for endogenous glycan binding proteins (2). They regulate cell physiology *via* interactions with proteins in their own membranes and by interacting with glycan binding proteins on other cells or in the extracellular milieu.

Congenital disorders of ganglioside biosynthesis reveal the essential roles of gangliosides in human physiology (3). Subjects lacking a ganglioside-specific biosynthetic enzyme, GM3 synthase (*ST3GAL5* c.862C>T), suffer from somatic growth failure, gastrointestinal dysfunction, and severe cognitive and motor impairment. Although most survive beyond 25 years, none were able to engage in reciprocal speech, most had hearing and vision impairments, many suffered chronic seizures, and few were able to sit independently. The devastating breadth of this disorder raises questions reviewed here – what are gangliosides, where are they expressed, how do they support the normal functions of cells and tissues, and how do they impact human pathology. Advances in ganglioside functions in receptor and ion channel regulation, and their roles in cancer and neurodegenerative diseases, are among emerging and ongoing areas of research reviewed.

## Ganglioside structures, biosynthesis and distribution

**Figure 1:**
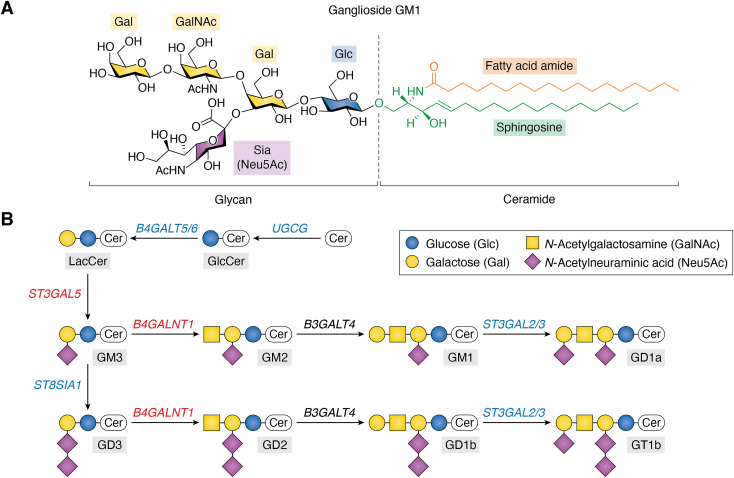
**Major mammalian gangliosides.***A*, ganglioside GM1 contains the gangliotetraose neutral core (Galβ1-3GalNAcβ1-4Galβ1-4Glcβ1-1′Cer) with a single sialic acid (Neu5Ac) in α2-3 linkage to the internal Gal. The ceramide structure is variable; the ceramide shown (d18:1/18:0) is abundant in the brain. *B*, eight major gangliosides in mammals and their biosynthesis starting from ceramide. Glycosyltransferase genes for each step are shown in in italics. Gene names in *red* designate those mutated in human congenital disorders of ganglioside biosynthesis. In addition to these, genes names in blue designate those mutated in KO mice. Structures are shown using the Symbol Nomenclature for Glycans (16).

Gangliosides are sialic acid bearing glycosphingolipids (Fig. 1). They are built on a ceramide lipid core composed of a long-chain amino alcohol, sphingosine, with its amino group in N-acyl linkage to a fatty acid, which is most often fully saturated. Their glycans are synthesized by glycosyltransferases that add a sugar first to ceramide then to the growing glycan chain one at a time (4). Some glycosyltransferases are specific for glycolipid biosynthesis while others are shared by glycoproteins. Gangliosides vary in their glycans and their ceramide structures. They are found in all mammalian cells and tissues in varying amounts and with tissue-specific structural distributions (5). Among human tissues, they are most abundant in brain grey matter (∼3 mg/g fresh weight (6, 7)) with sharply lower concentrations in other tissues (∼0.01–0.3 mg/g fresh weight). In human brain, gangliosides are the most abundant sialoglycans, representing >75% of the total bound sialic acid (8).

The smallest ganglioside carries just two sugars, Neu5Acα2-3Galβ1-1′Cer (9), and is the only native mammalian ganglioside based on a galactosylceramide core. All other mammalian gangliosides are based on the lactosylceramide core Galβ1-4Glcβ1-1′Cer, the simplest of which is GM3 (Neu5Acα2-3Galβ1-4Glcβ1-1′Cer), a common ganglioside in many human cells and tissues. Larger gangliosides are often based on the gangliotetraose neutral core (Galβ1-3GalNAcβ1-4Galβ1-4Glcβ1-1′Cer), but those based on the lacto, neolacto, and globo series glycosphingolipids are well established (10). Among the largest gangliosides are terminally sialylated poly-N-acetyllactosamine gangliosides (11, 12), some of which carry up to 18 sugars. Ganglioside expression is tissue-specific. For example, in mammals GM3 represents >50% of all gangliosides in major organs including the lung, liver, stomach, intestine and kidney (13). In contrast, GM3 expression is a very minor substituent in brain (13), where more complex ganglio-series gangliosides (GM1, GD1a, GD1b, GT1b) predominate (8).

Systematic ganglioside nomenclature is cumbersome, which led to broad adoption of the non-systematic nomenclature of Svennerholm (14, 15) for the most abundant ganglio-series gangliosides (Fig. 1). Svennerholm nomenclature embodies the letter “G” for the ganglio-series neutral core (up to 4 sugars) followed by a letter designating the number of sialic acids (M, D, T for mono, di, tri), a code number for the length of the neutral core, and a letter (a, b, c) designating the positions of sialic acids. Thus, GT1b is a trisialo ganglioside built on the gangliotetraose neutral core and having two of its three sialic acids on the internal galactose (Fig. 1). This review focuses mostly on the most abundant mammalian gangliosides in Figure 1 using their Svennerholm (14) and Symbol Nomenclature for Glycans (16) designations.

### Gangliosides in membrane structure

Gangliosides are found primarily in the outer leaflet of the plasma membrane, but are not uniformly distributed in the lateral plane of the fluid membrane. Like other sphingolipids, the ceramide lipid moiety of gangliosides typically has a long, saturated sphingosine terminus and a long fully saturated fatty acid amide (Fig. 1). Such ceramide lipid tails are tight-packing in the lateral plane, contributing to a “liquid-ordered” (Lo) membrane domain, which contrasts with most phospholipids that contribute to a more “liquid-disordered” domain (Ld). Evidence indicates that sphingolipids, including gangliosides, spontaneously organize laterally in the plasma membrane, equilibrating dynamically between more hydrophobic lateral assemblies – lipid rafts – and the bulk membrane (17). Lipid rafts can be detected in model and native membranes, and can be experimentally separated from bulk membranes to allow biochemical characterization. Partitioning into lipid rafts concentrates gangliosides when compared to the bulk membrane resulting in a local higher density with enhanced binding of select cations and ganglioside-specific binding proteins (18, 19, 20, 21). In addition, ganglioside neutral sugars and ceramide form hydrogen bonds with other membrane molecules at the lipid-water interface thermodynamically favoring raft formation (22, 23, 24). Selective interactions of gangliosides with other membrane lipids and proteins contribute to discrete lipid raft constituents and properties.

Giant unilamellar vesicles containing coexisting Lo and Ld lateral phases have been useful models of lipid rafts (25, 26). Lipid-lipid interactions in model systems reveal highly specific contributions of gangliosides to the separation of lateral domains and to the stability of Lo nanodomains (20, 26). Incorporation of GM1 as a prototypical ganglioside into membranes with native levels of outer leaflet cholesterol (27) results in increased stability (line activity) at Lo/Ld boundaries (28). Thus, gangliosides influence lateral lipid phase separation, which in turn affects their own functions as well as that of other lipid raft resident molecules.

Using multiple complementary methods, proteins such as flotillin, caveolin and Thy 1 are consistent raft markers while others, such as transferrin receptor, segregate to the bulk membrane phase (29, 30, 31, 32). Association of gangliosides with lipid rafts is further demonstrated by their co-isolation during physical separation of rafts from the bulk membrane in density gradient ultracentrifugation, where raft-resident proteins are ganglioside-associated, while others reside in ganglioside-poor membrane isolates. In some cases, the recruitment of specific proteins to rafts is transient precisely due to their ganglioside-binding. Ganglioside GM3 interacts with insulin receptor (IR) *via* binding between the negatively charged sialic acid of GM3 and a positively charged lysine residue in the juxtamembrane region of the IR (33, 34). An increase in GM3 in ganglioside-enriched lipid rafts prevents the functional association of IR with caveolin-1, causing impaired insulin signaling and insulin resistance that correlates with the concentration of GM3 (23, 33, 35). In this case, a ganglioside transiently directs the molecular and biophysical organization of the membrane through orchestrating specific ganglioside-lipid and ganglioside-protein interactions.

### Mechanisms of ganglioside-mediated regulation

**Figure 2:**
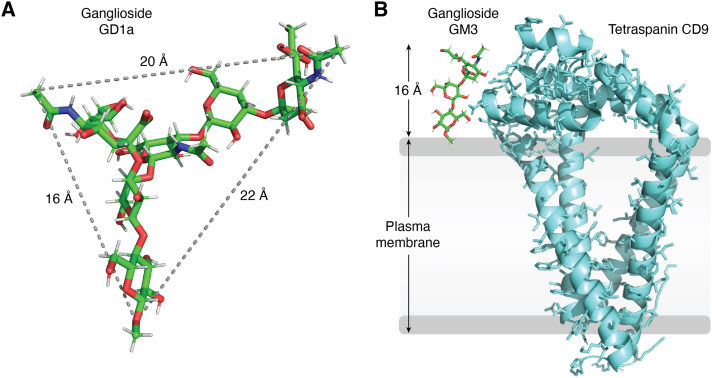
**Ganglioside size.***A*, ganglioside GD1a glycan with glucose at the bottom (normally attached to ceramide, not shown) and branched sialic acids at far left and right of the structure. *B*, GM3:tetraspanin size comparison. Energy-minimized GM3 (all-atom explicit solvent molecular dynamics simulation (205)) is shown extending from the plasma membrane (*gray*) in comparison with tetraspanin CD9 (*cyan*) with which it functionally associates to regulate integrin-growth factor signaling (206, 207).

Gangliosides orient at cell surfaces with their ceramide lipids deeply embedded in the bilayer of the plasma membrane and their glycans extending outward into the extracellular space (1). Many ganglioside glycans extend ∼16 to 22 Å above and 9 to 20 Å across the cell surface (Fig. 2), sweeping out considerable volume (36) and carrying a variety of molecular determinants that support specific interactions with other molecules at the cell surface and in the extracellular milieu. In addition, gangliosides associate in lipid rafts (see above) to form ganglioside-enriched islands on the plasma membrane. The physicochemical properties of gangliosides, *per se*, represent a driving force for membrane shape and for lateral molecular associations in the outer leaflet of the plasma membrane (36).

In addition to their physicochemical actions on membranes, gangliosides regulate cell functions by specifically binding to and modulating the functions of proteins (2, 37). This occurs in two fundamental orientations (38, 39). Gangliosides bind laterally to proteins embedded in their own membrane (*cis* interactions) and modulate their activity. In addition, ganglioside glycans bind to glycan-binding proteins on apposing cells (*trans* interactions) to mediate cell-cell recognition and modulate cell physiology. Such *trans* interactions may be facilitated by the lateral mobility of gangliosides and their propensity to cluster in lipid rafts, which may enhance affinity by multivalency. Examples of both *cis* and *trans* mechanisms of ganglioside-mediated molecular and cell regulation are well established, and in some cases may occur simultaneously to link extracellular recognition to intracellular signal transduction. Beyond *cis* and *trans* molecular interactions, gangliosides act as cell surface receptors for certain soluble proteins. This is best exemplified by soluble bacterial toxins such as cholera toxin and tetanus toxin (40), each of which requires a different ganglioside structure to intoxicate different cell types.

### Ganglioside recognition by proteins on apposing cells and in the extracellular milieu

#### Ganglioside as receptors for bacterial exotoxins

The virulence of some human bacterial pathogens is associated with bacterial secretion of protein toxins (bacterial exotoxins) that bind to human cells and modulate their activity (41). Several bacterial exotoxins bind to gangliosides – alone or in combination with other cell surface molecules - as their entry point to human cells (Table 1).

**Table 1:** Bacterial exotoxins and their ganglioside receptors

Bacteria	Toxin	Gangliosides	Molecular Target	Pathology
*C. tetani*	TeNT	GT1b, GD1b > GM1	VAMP	Spastic paralysis
*C. botulinum*	BoNT/A	GT1b > GD1a, GD1b	SNAP-25	Flaccid paralysis
	BoNT/B	GT1b > GD1a > GD1b	VAMP	Flaccid paralysis
	BoNT/D	GD2 > GT1b, GD1b	VAMP	Flaccid paralysis
*V. cholerae*	Ctx	GM1	Gα_S_	Diarrheal disease
Enterotoxigenic *E. coli*	LT-I	GM1	Gα_S_	Diarrheal disease
	LT-IIa	GD1b > GD1a > GM1	Gα_S_	Diarrheal disease
	LT-IIb	GD1a > GD1b	Gα_S_	Diarrheal disease

Data selected from Zuverink and Barbieri (40) and Poulain *et al.* (52).

TeNT, tetanus neurotoxin; BoNT, botulinum toxin; Ctx, cholera toxin, LT, heat-labile enterotoxin.

The link between gangliosides and bacterial exotoxins was first reported for tetanus toxin (42). In the late 19th century, a pathogen that causes tetanus (spastic paralysis), *Clostridium tetani*, was isolated from soil. Its culture supernatant was found to contain a secreted toxic protein, tetanus toxin. When tetanus toxin was mixed with brain homogenate – especially from brain grey matter – the toxin was cleared from solution upon centrifugation. The toxin “fixing” substance (receptor) was resistant to extraction from brain with acetone or ether, but susceptible to extraction with aqueous ethanol (43). Decades later, these studies were revisited after Klenk discovered gangliosides as components of brain grey matter with properties similar to those of the tetanus toxin receptor (44). Purified brain gangliosides were found to be potent toxin binding components (42).

**Figure 3:**
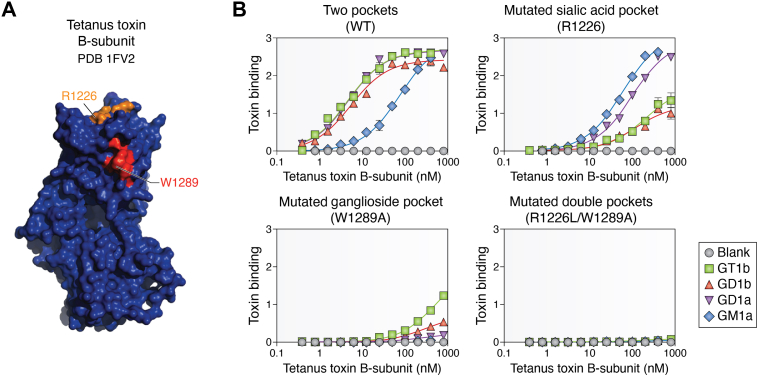
**Tetanus toxin binding to gangliosides.***A*, structure of the tetanus toxin binding B-subunit (PDB entry 1FV2) with dual ganglioside binding pockets highlighted in *orange* and *red*. *B*, quantitative binding of tetanus toxin receptor binding domain (WT) to immobilized gangliosides. Adjacent panels show binding of site-directed mutants R1226 L, W1289 A, and both. From Chen *et al.* (45) with permission.

After purification and structural determinations of gangliosides advanced, comparison of different gangliosides for their relative affinities to tetanus toxin followed (45, 46). Taken together, *in vitro* binding of tetanus toxin to immobilized gangliosides indicated broad specificity for major brain gangliosides, with highest affinity for GT1b and GD1b. This specificity was confirmed and extended *in vivo* using mice genetically engineered to lack selected ganglioside biosynthetic enzymes (47). Relative toxin susceptibility was quantified in WT and KO mice by measuring the time to death after intravenous injection of purified tetanus holotoxin (48). Mice with disrupted *B4galnt1* (Fig. 1) express abundant truncated gangliosides GM3 and GD3, but lack major brain gangliosides. The susceptibility of *B4galnt1*-null mouse to tetanus toxin-induced death was reduced 500-fold compared to WT mice (49). In comparison, *St8sia1*-null mice express abundant GM1 and GD1a, but lack b-series gangliosides GT1b and GD1b. *St8sia1*-null mice were ∼50-fold less susceptible to tetanus toxin-induced death compared to WT (50). Structural studies combined with site-directed mutagenesis (Fig. 3) provided a mechanism for this specificity (45), indicating two adjacent binding sites that contribute to optimal ganglioside binding. Mutating R1226 diminished GT1b and GD1b binding with no effect on GM1 binding; mutating W1289 diminished but didn’t eliminate ganglioside binding; and mutating both eliminated ganglioside binding. The data indicate synergistic binding *via* adjacent regions of the toxin receptor.

Tetanus toxin is representative of a larger family of related clostridial toxins with AB subunit structure in which the cell-binding B-subunit is essential to the subsequent toxic action of the enzymatic A-subunit. Whereas tetanus toxin causes spastic paralysis, structurally related botulinum toxins cause flaccid paralysis (51). This difference occurs because tetanus toxin is transported retrogradely from the neuromuscular junction to inhibitory neurons of the spinal cord, blocking inhibition, whereas botulinum neurotoxins remain at the neuromuscular junction and block contraction. Different *C. botulinum* serotypes secrete different toxins, each with the same overall structure and toxic mechanism but with distinct ganglioside binding specificities. Some serotypes bind preferentially to GT1b, others to GD2, and yet others to GM1 (52). Whereas mice lacking complex gangliosides (*B4galnt1*-null mice) are markedly (500-fold) less susceptible to tetanus toxin, their susceptibility to botulinum toxins is reduced only 12-36-fold depending on the serotype (49). *St8sia1*-null mice, lacking b-series gangliosides, retain WT susceptibility to botulinum toxin, unlike their reduced susceptibility to tetanus toxin (50). One can conclude that the ganglioside binding function of clostridial toxins evolved to enhance synaptic protein binding with different binding specificities and functional impacts.

**Figure 4:**
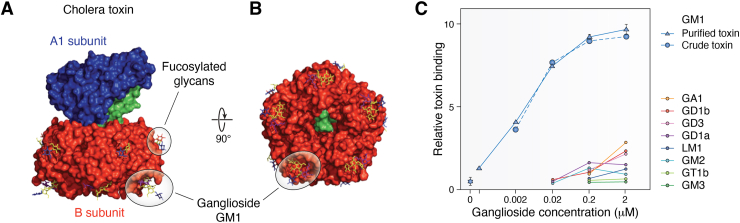
**Cholera toxin structure and ganglioside binding.***A*, side view of the holotoxin. The pentameric B-subunit (*red*) binds ganglioside *via* a site on its lower face. Pentameric binding to GM1 on the intestinal epithelial cell surface is followed by insertion of the toxic A1-subunit (*blue*) through the membrane where it ADP-ribosylates the α-subunit of the stimulatory G protein, activating adenylate cyclase to induce water efflux and diarrhea. *B*, view of the toxin from the *bottom* showing the five B-subunits each engaging GM1. *C*, quantitative measurement of the binding of cholera holotoxin to different adsorbed gangliosides. *A* and *B*, from Kumar and Turnbull (53); *C* from Holmgren *et al.* (46).

Compared to the clostridial toxins, cholera toxin is highly specific for one major ganglioside, GM1. Cholera toxin is representative of a family of toxins with AB5 structure in which a single toxic A-subunit associates with a pentamer of ganglioside-binding B-subunits (Fig. 4, (40, 53)). Coordination of the pentameric binding sites results in very high ganglioside avidity. Purified GM1, when premixed with cholera holotoxin, potently inhibited its ability to induce intestinal water efflux in rabbit intestine, a gold standard for its toxicity (54, 55). Cholera toxin B-subunit also has a fucose binding domain (distinct from its GM1 binding domain, Fig. 4*A*) that may independently support cholera toxicity (56). Ganglioside binding is shared by structurally related *Escherichia. coli* heat labile enterotoxins, which have overlapping but less stringent ganglioside specificities (Table 1, (57)).

Beyond their roles as receptors for soluble bacterial toxins, gangliosides support binding by select viruses (58) and pathogenic bacteria (59). Evolutionary persistence of gangliosides in the face of pathogen targeting suggests their roles in physiological protein recognition. Examples of endogenous ganglioside binding proteins follow.

#### Gangliosides in cell-cell recognition

Ganglioside binding proteins in vertebrates support cell-cell recognition and regulate cell behaviors essential for optimal physiological function. Gangliosides are functional ligands for select members of sialic acid-binding Siglec family (60) and Selectin family (61) members involved in cell-cell interactions that support optimal nervous system and immune system functions respectively.

**Figure 5:**
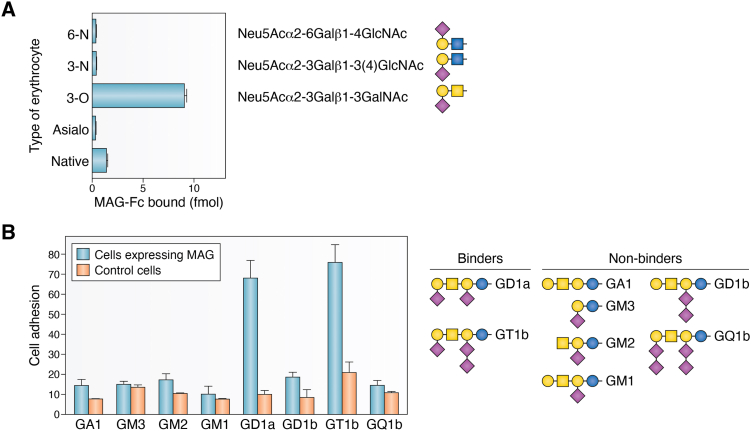
**Myelin-associated glycoprotein (MAG, Siglec-4) glycan and ganglioside binding specificity.***A*, MAG binding to erythrocytes bearing sialoglycans with different linkages. Human erythrocytes (Native) were treated with sialidase (Asialo) and portions re-sialylated by incubating with CMP-Neu5Ac and purified mammalian sialyltransferases (6-N, 3-N, and 3-O) that regenerate structures shown to the right of each bar. Native and modified erythrocytes were incubated with MAG-Fc chimera and binding determined. Data from (208). *B*, selective adherence of cells ectopically expressing MAG or control cells to microwell-immobilized gangliosides. Data from (209). MAG, myelin-associated glycoprotein.

There are 14 members of the human sialic acid-binding immunoglobulin-like lectin family (Siglecs). They have varied sialoglycan binding specificities with different Siglecs binding preferentially to α2-3, α2-6 or α2-8-linked sialic acids with affinity also dependent on the underlying neutral glycans (60). The first paper to report what became known as Siglecs reported sialic acid binding and structural similarity of 3 proteins, sialoadhesin (Siglec-1), CD22 (Siglec-2) and myelin-associated glycoprotein (MAG, Siglec-4). They found that MAG bound most robustly to Neu5Acα2-3Galβ1-3GalNAc termini (Fig. 5), the trisaccharide terminus of two major vertebrate brain gangliosides, GD1a and GT1b (Fig. 1). Direct studies using purified gangliosides revealed that GD1a and GT1b support MAG binding, whereas closely related gangliosides lacking that terminus (GM1, GD1b, GQ1b) do not (Fig. 5).

The physiological and pathological significance of MAG-ganglioside binding was confirmed in mice and humans (62, 63). MAG is expressed by myelinating cells that wrap nerve axons with multi-layered myelin membrane sheathes required for rapid nerve conduction (64). MAG is found on the innermost myelin sheath in close juxtaposition to the axon surface (65) where it supports axon integrity, stability and structure (66, 67). *Mag*-null mice and those engineered to lack the N-acetylgalactosaminyltransferase responsible for initiating the terminal MAG-binding trisaccharide on gangliosides (*B4galnt1*-null) share the same phenotype, including progressive motor behavioral deficits downstream of profound axonal degeneration (62). Notably, human subjects with congenital mutations in the same gene (*B4GALNT1*) suffer progressive spastic paraplegia, as do rare individuals carrying a single amino acid mutation of the key MAG arginine (R118) required for ganglioside binding (68). Structural analysis indicates that MAG-ganglioside binding across the myelin-axon interface draws the membranes closer together (69), initiating axon-supportive signaling and stabilization required for long term effective nerve conduction. As discussed in detail below, in addition to motor deficits, human subjects with congenital disorders of ganglioside biosynthesis invariably suffer cognitive deficits. Understanding the mechanisms of those functions of gangliosides await further investigation.

Gangliosides are expressed on all human cells with distinct cell-to-cell variations in ganglioside structural repertoires. Among human immune cells, lymphocytes and monocytes express high levels (70–90%) of GM3 (Neu5Acα2-3Galβ1-4Glcβ1-1′Cer) and lower levels of neolactoseries gangliosides with the general linear sequence Neu5Acα2-3[Galβ1-4GlcNAcβ1-3]_n_Galβ1-4Glcβ1,1′Cer (70). Human granulocytes express the same structures but flipped percentages; ∼10% GM3 with the balance primarily neolactoseries gangliosides. In neutrophils, neolactoseries gangliosides include a series of terminally sialylated and variably fucosylated poly-N-acetyllactosamine structures (11), some of which are implicated in neutrophil migration to sites of infection (12).

**Figure 6:**
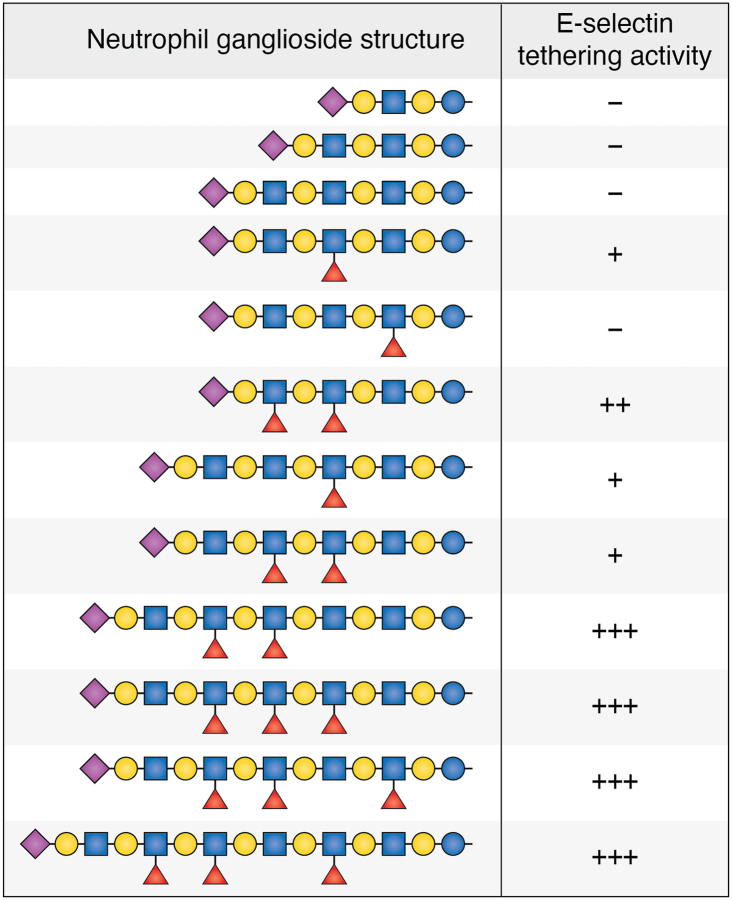
**Human neutrophil gangliosides support E-selectin tethering activity** (12). Monosialogangliosides were extracted from 10^7^ freshly isolated human neutrophils, purified by high pressure liquid chromatography, analyzed by tandem mass spectrometry, and tested for their ability to support tethering by intact cells engineered to express E-selectin. Structures of the predominant species are presented using symbol nomenclature for glycans (16) along with their relative E-selectin dependent tethering activity.

Among human sialic acid binding proteins expressed on cell surfaces are the C-type lectin subfamily of Selectins, comprised of E−, L-, and P-Selectin, that regulate leukocyte adhesion to the vascular wall (61). E− and P-selectin are expressed on the luminal plasma membrane of vascular endothelial cells in response to nearby infection. They then bind to sialoglycans constitutively expressed on neutrophils, initiating cell adhesion and neutrophil extravasation into the adjacent tissue to fight infection. Selectins bind to neutrophil cell surface glycans with appropriately spaced sialic acid and fucose residues. The sialoglycan ligand for P-selectin is well established as a glycoprotein (PSGL-1) on both mouse and human neutrophils (71). Treatment of freshly isolated human and mouse neutrophils with protease, as expected, removed PSGL-1 and the cells from both species lost their ability to bind P-selectin. Of interest, similar protease treatment resulted in loss of E-selectin binding to mouse neutrophils, but not to human neutrophils (72). This led to the hypothesis that fucosylated gangliosides, similar to those first identified on human myelogenous leukemia cells (11), might be functional human E-selectin ligands on human neutrophils (12). Extraction of monosialogangliosides from human neutrophils revealed a series of sialylated fucosylated structures (Fig. 6). Fucosylation density increased as the length of the glycan increased, and the longest most highly fucosylated structures potently supported E-selectin-mediated cell tethering. This series of large gangliosides, containing up to 18 saccharides may be essential to initiating neutrophilic inflammation in humans.

### Lateral association of gangliosides with membrane proteins

#### Neurotransmitter receptors

Gangliosides interact with both major classes of neurotransmitter receptors, ionotropic and metabotropic. These interactions are conveyed through direct binding, by acting as co-receptors for their ligands, and by regulation of receptor trafficking and recruitment to specific synaptic domains.

Ionotropic neurotransmitter receptors are ligand-gated ion channels and their activation results in opening ion channels through which different ions will flow, *e.g.,* Na^+^, K^+^, or Cl^-^. GM1 co-segregates with AMPA receptors (AMPARs) in lipid rafts (73) as well as directly bind GluA2 subunit-containing glutamate AMPARs (74) that mediate fast excitatory neurotransmission (75). The action on AMPARs is a fitting illustration of the biform nature of specific ganglioside species, since another ganglioside, GT1b, binds the AMPAR-trafficking complex which is responsible for the internalization of AMPAR (74). Hence, the binding of GM1 and GT1b to either the AMPAR, recruiting it to specific concentrated synaptic signaling domains, or to AMPAR internalization complexes leading to the removal of AMPAR from the synaptic membrane, has a direct effect on the amount of AMPAR present in the synaptic membrane (74). GQ1b facilitates the activation of another type of ionotropic glutamate receptors, N-methyl-D-aspartate receptors(76). This ganglioside enhances the NMDA signaling pathway resulting in increased BDNF expression (77). Again, a different ganglioside, GM1, enhances the activity of the BDNF receptor, TrkB (78), which shows that different gangliosides can act on different components of the same signaling pathway. In addition to the effect on AMPARs and N-methyl-D-aspartate receptors binding glutamate, the role of gangliosides in glutamate release has been demonstrated in cell lines with depleted gangliosides (79). Specifically, ganglioside depletion from the membrane leads to decreased depolarization-evoked glutamate release (80, 81). Furthermore, changes in ganglioside expression disrupt the glutamatergic synapse function, possibly since the negative electric fields surrounding gangliosides and consequently lipid rafts contribute to the control of the flow of glutamate in the tripartite synapse (21). Exogenous administration of GM1 was reported to protect from glutamate-excitotoxicity (82), further underlying the elaborate relationship between gangliosides, glutamate and glutamate receptors.

The negative charges of ganglioside sialic acids are also implicated in dopamine uptake. Specifically, GM1 was found to bind neuronal dopamine transporter, a membrane protein responsible for re-uptake of dopamine from the synaptic cleft into the presynaptic neuron. GM1 modulates the kinetic parameters of the transporter consequently modifying the amount of dopamine in the synaptic cleft (83).

GM1 also has an effect on serotonin neurotransmission through the metabotropic 5-HT1 serotonin receptor. 5-HT1 is a G protein-coupled receptor (GPCR) with a sphingolipid binding domain and GM1-specific interaction sites were revealed by molecular dynamics simulations (84) and synergistic co-binding with cholesterol (85, 86). Gangliosides and cholesterol not only bind the serotonin receptor and induce conformational changes, but aid in solubilization of serotonin aggregates in the synaptic cleft, funneling serotonin to the receptor and assisting in serotonin signaling (86). Ganglioside effects on selected neurotransmitter systems are summarized in Table 2.

**Table 2:** Selected ganglioside effects on neurotransmitter receptors and neurotransmission-related proteins

Receptor type	Receptor/protein	Primary endogenous ligand	Receptor/protein role	Ganglioside (effect)	Reference
Ionotropic	AMPAR	Glutamate	Fast excitatory neurotransmission through influx of mostly Na^+^ ions into the postsynaptic neuron, depending on subunit composition influx of Ca^2+^ ions	GM1 (↓)	(74)
	AMPAR-trafficking complex	AMPAR	Internalization of AMPAR	GT1b (↑)	(74)
Ionotropic	NMDAR	Glutamate + co-agonist	Excitatory neurotransmission through the influx of primarily Ca^2+^ followed by Na ^+^ ions	GQ1b (↑)	(76, 77)
	Neuronal dopamine transporter	Dopamine	Uptake of dopamine from the synaptic cleft back into the presynaptic neuron	GM1 (↑)	(83)
Metabotropic	5-HT1	Serotonin	Inhibitory neurotransmission through a local decrease in cAMP concentrations and modulated downstream effects	GM1 (↑)	(84, 85, 86)

#### Ion transport

Gangliosides can affect intracellular ion concentrations by modulating ionotropic neurotransmitter receptors (Table 2), but also through affecting ion pumps and channels (87). This fact was established in studies probing for ganglioside-interacting partners (88), but also in focused investigations of Na^+^, K^+^-ATPase (NKA), plasma membrane calcium ATPase (PMCA) and calcium channels.

NKA activity is dependent on its lipid environment; the enzyme activity of this ubiquitous essential membrane pump is modulated by the major plasma membrane lipids phospholipids and cholesterol (89, 90, 91, 92, 93). More recent findings reveal that gangliosides also modulate NKA activity, if not by direct binding, then through ensuring the membrane microlocation of the pump either in the pumping or non-pumping pool within the membrane (94, 95). Thermally induced NKA redistribution within the membrane in mouse cortical homogenates is accompanied by reshuffling of specific gangliosides. GM1’s distribution in membrane domains follows the NKA protein distribution; a shift from lipid rafts to the bulk domain for both GM1 and NKA is evident with membrane remodeling. On the other hand, the activity of NKA correlates with the abundance of GD1a, GD1b and GT1b in distinct membrane domains, gangliosides with a richer topology than GM1 (94). These findings indicate that discrete ganglioside environments regulate NKA function. Experiments with exogenously added gangliosides performed in various biological samples routinely reveal that NKA activity is affected by the addition of gangliosides (95). In most studies, GM1 is found to increase NKA activity (96, 97, 98), however some studies in different species and biological sample types report conflicting results (99, 100), pointing out the necessity to thoroughly evaluate the interplay between NKA and gangliosides.

PMCA is an ATP-hydrolyzing pump critical for maintaining intracellular Ca^2+^ concentrations. Due to its importance in calcium homeostasis, the lipid environment of PMCA has been investigated in numerous studies focusing on different lipid membrane constituents (101, 102). Differential effects of gangliosides on PMCA were established: mono-sialogangliosides, most notably GM1, appear to inhibit PMCA, while poly-sialogangliosides stimulate its activity (103, 104). PMCA has several isoforms, expressed in different cell types and different membrane domains. These membrane-domain subtypes, intrinsically marked by specific ganglioside content, have different roles in calcium signaling (105). However, the nature of the exact effect of gangliosides on PMCA is not clear. A compelling finding illustrating an additional level of complexity of the gangliosides-Ca^2+^ homeostasis nexus is that neuroplastin, an essential auxiliary subunit of selected PMCA isoforms, is also heavily influenced by gangliosides (106, 107, 108). GM1-containing lipid rafts stabilize PMCA-neuroplastin complexes, and blocking GM1 with specific antibodies results in delayed calcium restoration of electrically evoked calcium transients in the soma of hippocampal neurons (106). Since the controlled exchange of Ca^2+^ between the cell and extracellular space is a prerequisite for normal neuronal function, the influence of GM1 on calcium homeostasis is of physiological and pathological interest.

GM1 also modulates different types of voltage-gated calcium channels (109). Specifically, GM1 inhibits L-type calcium channels but enhances N-type and P-type calcium channels (110, 111, 112). L-type calcium channels are primarily found in cardiac and smooth muscles, while N/P-type calcium channels are predominantly expressed in the nervous system and are involved in neurotransmitter release. Since gangliosides influence Ca^2+^ homeostasis intracellularly as well, *e.g.,* through binding Na^+^/Ca^2+^ exchanger (NCX) in the nuclear envelope (NE) (113) and causing consequent release of Ca^2+^ ions from the nucleoplasm to the endoplasmic reticulum, they appear to be involved in cell-wide regulation of calcium homeostasis (114).

Even though there is a wealth of empirical evidence that gangliosides exert strong and quantifiable effects on several ion pumps and channels, influencing cellular ion homeostasis, the details of their interactions remain an enigma. Studies aiming at investigating binding sites, affinities and kinetic parameters are necessary to fully describe the role of gangliosides in maintaining ion balance of the cell.

#### Intracellular gangliosides

Although gangliosides predominantly reside in the plasma membrane, that localization is not exclusive. Apart from the Golgi complex where they are synthesized, and lysosomes where they are degraded (8, 115), lower amounts of gangliosides have been reported on the NE, at ER-mitochondria contact sites, and trafficked to mitochondria (116).

GM1 and GD1a are detected on the NE, where GD1a may be a precursor for GM1 through the action of neuraminidases – Neu3 on the NE inner membrane and Neu1 on the outer membrane (117, 118). GM1 appears to bind to the NCX of the NE, increasing its activity (113). The complex between GM1 and NCX results in transfer of Ca^2+^ ions from the nucleoplasm to the NE lumen and consequently to the ER, directly participating in cellular Ca^2+^ homeostasis. Deficiency of nuclear GM1 correlates with vulnerability of cells to apoptosis, perhaps as a result of perturbed Ca^2+^ equilibrium (118).

GM1 has also been reported to contribute to epigenetic regulation by binding acetylated histones and influencing expression of glycosyltransferase genes (119, 120). Histones H3 and H4 on the *B4galnt1* gene promotor may be targets of this pathway, resulting in recruitment of trans-activation factors and epigenetic up-regulation of gene expression (121). Whether direct or indirect, reports of beneficial effects of intravenous GM1 administration in humans with Parkinson’s disease correlated with epigenetic hypermethylation of cAMP responsive element binding protein 5 (CREB5), which regulates the expression of dopaminergic neuron-related genes (122). GD3 was reported to effect histone acetylation when translocated from cytosol to the nucleus. That translocation correlates to rapid H1 histone phosphorylation, a post-translational modification favoring apoptosis through regulation of gene expression (123).

In addition to the nucleus, gangliosides were reported at mitochondria-associated ER membranes, the contact sites between the ER and mitochondria that control Ca^2+^ flux between these organelles, where GD3 was postulated to be involved in autophagosome formation (124). In an animal model of GM1 storage disease, evidence for GM1 accumulation in the lipid raft fraction of mitochondria-associated ER membrane correlated with a Ca^2+^-mediated ER stress response and induction of a mitochondrial apoptotic cascade (125). GM1, which can be pro-apoptotic or anti-apoptotic depending on its local concentration, may serve as a checkpoint integrating apoptotic signals and regulating cell apoptosis (124, 126, 127).

The underlying motif for the findings on intracellular gangliosides is that they configure microdomains of organelle membranes: nuclear membranes, mitochondria-associated ER membranes, and mitochondrial membranes, where they influence protein activity and gene expression (128, 129).

### Impacts of gangliosides on human physiology and pathology

#### Congenital disorders of ganglioside biosynthesis

The varied physiological functions of gangliosides are revealed by human congenital disorders resulting from mutations of two biosynthetic genes specific for their biosynthesis (see Fig. 1), *ST3GAL5* (GM3 synthase) and *B4GALNT1* (3, 63, 130). Although rare, dozens of affected subjects have been reported, providing insights into the functions of gangliosides in humans. Most compelling are subjects with *ST3GAL5* gene mutations, the outcomes of which are profound (131). Detailed histories of 50 subjects of North American Amish ancestry (3) with the same genetic variation (c.862C>T) and 16 subjects representing six other genetic variants (131) revealed very similar clinical outcomes. All of the subjects suffered from severe to profound intellectual disability. None attained language use. Most lacked eye contact and suffered hearing loss. Seizure and dyskinetic movements were common and motility was severely impaired in all subjects. Gastrointestinal problems (reflux, constipation) were common (80–90%) as were eating difficulties. Sleep problems combined with persistent irritability often resulted in subject management challenges requiring medical intervention. The diverse and profound outcomes of *ST3GAL5* mutations limit mechanistic interpretation, instead suggesting that gangliosides may function in multiple molecular regulatory roles. In contrast, *St3gal5* gene deletion in mice resulted in markedly less severe outcomes, perhaps due to compensatory biosynthesis of alternate ganglioside structures (35, 132).

Congenital mutations of the *B4GALNT1* gene, which is further down the biosynthetic pathway to major brain gangliosides (Fig. 1) result in serious but less severe outcomes compared to subjects with *ST3GAL5* mutations (133, 134). Depending on the specific mutation, subjects present with gait disorders in the first decades of life, and suffer progressive motor deficits predominantly in the lower limbs that over decades may result in mobility loss (63). Mutations in *B4GALNT1* are clinically classified as a form of hereditary spastic paraplegia, a broad set of genetic disorders downstream of any of dozens of genes that drive neuromuscular function. *B4GALNT1* is designated as spastic paraplegia gene 26 (SPG26). hereditary spastic paraplegia’s are marked by progressive muscle weakness, particularly in the lower limbs. In the case of SPG26, subjects have been characterized as suffering from peripheral neuropathy predominantly of the axonal type. They also suffer intellectual disability that ranges from mild to severe (IQ 50–70), some also presenting with seizures.

Molecular mechanisms of the disorder are informed by study of a mouse model of *B4galnt1* disruption. Homozygous null mice display gait and motor functional disorders at 3 to 6 months that progresses to loss of hindlimb function in older mice (62). These deficits correlate with peripheral neuropathy of the axonal type downstream of disrupted axon-myelin interactions, findings fully consistent with SPG26 subjects. Data support a functional association of complex gangliosides (GD1a, GT1b) lost in *B4galnt1* mutants with MAG. Notably, the phenotype of *B4galnt1* null mice was remarkably similar to mice lacking MAG, supporting myelin destabilization *via* ganglioside-MAG interactions as the mechanism for spastic paraplegia in SPG26 subjects (62).

Five genes responsible for enzymes on the biosynthetic pathway to major brain gangliosides (Fig. 1) have been knocked out in mice, *St3gal5*, *St8sia1*, *B4galnt1*, *St3gal2*, and *St3gal3* (47, 135, 136). In each case, blocking the biosynthetic pathway resulted in build-up of the ganglioside species behind the block such that total brain ganglioside concentration remained stable. In mice, disrupting *St3gal5* results in equivalent buildup of what normally are rare gangliosides that lack the sialic acid on the internal galactose including GM1b (Neu5Acα2-3Galβ1-3GalNAcβ1-4Galβ1-4Glcβ1,1′Cer) and GD1α (Neu5Acα2-3Galβ1-3[Neu5Acα2-6]GalNAcβ1-4Galβ1-4Glcβ1,1′Cer); disrupting *St8sia1* in buildup of GM1 and GD1a; and disrupting *B4galnt1* in buildup of GM3 and GD3 consistent with each enzyme’s role in complex ganglioside biosynthesis (Fig. 1). To what extent this happens in human subjects is unknown, due to limited tissue access. In mice, both *St3gal2* and *St3gal3* can contribute to terminal sialylation of GD1a and GT1b (136). Disrupting both genes resulted in buildup of the precursors GM1 and GD1b. Disease-associated congenital mutations in *ST3GAL2* have not been reported, whereas mutations in *ST3GAL3* resulted in intellectual disability including West Syndrome (137, 138). The extent to which these deficits are ganglioside and/or sialoglycoprotein driven is not known.

### Ganglioside dysregulation in cancer

Dysregulation of ganglioside expression was initially reported in melanoma cells (139) and other cancers of ectodermal origin such as neuroblastoma, glioblastoma, and breast cancer (140, 141, 142). Subsequently, alterations in ganglioside expression were documented in tumors of mesodermal (143) and endodermal origin (144, 145) implicating changes in ganglioside biosynthesis as a common factor in cancer progression (146). Some gangliosides have garnered considerable attention as cancer targets (147), including (depending on the cancer) GD3, GD2, GM2, and GM3 (structures, Fig. 1). Some cancer cells overexpress gangliosides that were expressed at earlier developmental stages. For example, during mammalian brain development ganglioside expression is dominated by shorter, precursor structures (GM3, GD3) over mature gangliotetraose structures (GM1, GD1a, GD1b, GT1b) by a ratio of 4:1 (148). At birth, that ratio flips to 16:1 with mature structures dominating. By comparison ganglioside expression in human neuroblastoma, although variable, favors precursor structures by a ratio of nearly 2:1 (149). In contrast, mammary gland cells normally contain only small amounts of complex gangliosides whereas breast carcinoma cells may activate an enzyme that adds a sialic acid to the GalNAc 6-hydroxyl of gangliosides (*e.g.,* GM1), resulting in the accumulation of α-series gangliosides, such as GD1α (149). Although deviations in expression patterns of gangliosides were noted in cancer (150), variability of ganglioside profiles among patients with the same tumor type (151) and differing ganglioside profiles between primary tumors and metastases (152) have limited the use of gangliosides in diagnostic applications. Variability in ganglioside expression among tumors is rooted in epigenetic regulation of the biosynthetic and catabolic enzymes, including glycosyltransferases and NEU3, a ganglioside-directed membrane sialidase (153). Furthermore, cancer-associated disruption of glycosylation often impacts glycoproteins, further contributing to glycan heterogeneity (154). The complexity of these alterations has been addressed by high-throughput methods, with the goal of tracking overall aberrant glycosylation for more precise diagnosis, prognosis, and therapy (154).

Dysregulation of ganglioside expression has been reported to regulate key aspects of cancer progression, including cell growth, cell adhesion, angiogenesis, and immune surveillance (153, 155). Gangliosides affect tumor proliferation *via* regulation of receptor tyrosine kinases (RTKs) such as epidermal growth factor receptor (EGFR), platelet-derived growth factor receptor, fibroblast growth factor receptor, c-Met (hepatocyte growth factor receptor), and IR in a context-dependent manner (156, 157). They modulate RTK receptor-ligand interactions, dimerization, and subcellular localization (157, 158). Additionally, through actions on transmembrane vascular endothelial growth factor receptors (VEGFR)-1 and VEGFR-2, gangliosides (GD1a and GM3 in particular) influence tumor angiogenesis (159, 160) that drives growth of solid tumors. A recent study indicates that cancer cells over-expressing the ganglioside-specific glycosyltransferase *ST8SIA1* (GD3-synthase) downstream of a p53 mutation protects them from apoptosis, enhancing tumor progression (161).

In addition to regulation of the cells on which they are expressed, gangliosides are also shed into the surrounding environment in the form of monomers, micelles, and extracellular vesicles (162, 163). Shed gangliosides can be immunosuppressive, in part *via* immune inhibitory sialic acid binding proteins (Siglecs) on immune cells (164). Some of the characteristic effects include inhibition of immune cell proliferation, activation, cytotoxicity, and/or production of pro-inflammatory cytokines by T cells, as well as decreased Ig production by B cells (165).

Gangliosides are of interest as targets of anti-cancer drug therapy, although therapeutic success to date has been limited (146). Dinutuximab, an antibody to GD2, enhances outcomes in pediatric high-risk neuroblastoma, with significant but manageable side effects (166). However, targeting gangliosides is complicated by mixed effects on tumorigenicity and immune modulation. Moreover, since gangliosides regulate diverse physiological processes, targeting cancer gangliosides can generate off-target effects. Gangliosides with low expression after development may be acceptable therapeutic targets, such as GD2, O-acetyl-GD2 (167, 168), and Fuc-GM1. An interesting therapeutic target is GM3(Neu5Gc), the ganglioside GM3 with an extra hydroxyl group on the N-acetyl moiety (N-glycolyl in place of N-acetyl) on the 5-carbon of its sialic acid. Humans don’t express the hydroxylase, common in other animals, that synthesizes N-glycolylneuraminic acid (Neu5Gc) (169), but can incorporate Neu5Gc from dietary sources such as red meat (170). Given the high metabolism of cancer cells, Neu5Gc uptake and incorporation is enhanced, and GM3(Neu5Gc) has been detected in breast cancer, melanomas, gastrointestinal tract tumors, among others, making it an intriguing therapeutic target (171).

Modulation of the ganglioside biosynthesis pathway *via* targeting glycosyltransferases may also offer therapeutic strategies (172). In some cases, increasing gangliosides may be therapeutic. Ganglioside GM3 inhibits RTKs including EGFR, fibroblast growth factor receptor and VEGFR (156). Valproic acid, an approved anti-epileptic drug and histone deacetylase inhibitor, was serendipitously found to increase GM3 synthesis (*via ST3GAL5*) and suppress proliferation of select cancer cells *in vitro* (173). To date, modulation of ganglioside expression *via* their biosynthetic enzymes has not reached the clinic.

Another potential therapeutic approach is generation of anti-ganglioside cancer vaccines. However, gangliosides generally have low immunogenicity, requiring chemical conjugation, structural modification or cell glycoengineering (174). In an encouraging preclinical study, GD2-specific human CAR-T cells developed from peripheral blood of glioblastoma patients were effective in clearing tumors from mice carrying human glioblastoma xenografts (175), offering an example of tailored anti-ganglioside therapies that might be developed therapeutically.

#### Gangliosides in neurodegenerative diseases

The three most common neurodegenerative diseases – Alzheimer’s (AD), Parkinson’s disease, and Huntington’s disease– share dysregulation of ganglioside expression in their pathophysiological profiles (Table 3) (116, 176). These diseases are marked by the aggregation and accumulation of toxic proteins – Aβ, α-synuclein, and mutated huntingtin (mHTT), respectively (177, 178, 179). Whether disease progression alters ganglioside expression and/or altered ganglioside expression contributes to disease progression remains to be established (180, 181). However, multiple lines of evidence support the hypothesis that gangliosides modulate oligomerization of toxic proteins (182, 183, 184, 185). Furthermore, *in vitro* and *in vivo* data report changes in gangliosides that precede toxic protein aggregation (186, 187, 188).

**Table 3:** Role of gangliosides in neurodegeneration

	AD	PD	HD
Misfolded protein	Aβ, hyperphosphorylated Tau (210, 211, 212)	α-synuclein (213)	huntingtin, mHTT (214)
Neurons affected	Cholinergic (192)	Dopaminergic (215)	GABAergic (194)
Affected brain region	Hippocampus/cortex	Substantia nigra pars compacta	striatum, cortex
Ganglioside expression (human)	↓GM1, GD1a, GD1b, GT1b (216, 217, 218) ↑GM2, GM3, GM4 (189) ↓GQ1bα and GT1aα (219)	↓GM1, GD1a, GD1b, GT1b ↑GM3 (190)	↑GD3 ↓total gangliosides (220)
Ganglioside biosynthetic enzymes	↓ Sialyltransferases (221)	↓B4GALNT1, ST3GAL2 (222)	↓ glycosyltransferases (220)
Peripheral ganglioside dysregulation	↑Fibroblast catabolism of GM1 ↑β-hexosaminidase/β-galactosidase ↑β-galactosidase in leukocytes (223)	↓ Gangliosides in serum (224) ↓ GM1 in heart and colon (225)	↓ gangliosides in patient’s fibroblasts (226)
Animal models with ganglioside dysregulation or ganglioside administration	Tg APP21 (rat) (227), 1XFAD/B4GALNT1^−/−^ (228) APP/PSEN1/ST8SIA1^−/−^ (229) 5XFAD/ST3GAL5^−/−^ (230) 5XFAD//UGCGF/F/THY-CreERT2//EYFP (231)	B4galnt1^−/−^ (188) MPTP model (232)	Yac128 (full length mHTT) (184) R6/1 (exon 1 mHTT) (226)
Effects of exogenous gangliosides – animal models and clinical trials	↓Apoptosis, ↓ fibril formation (233) ↑Cognitive functions (234), autophagy (235) ↓Oxidative stress (236)	↑Clearance and correct folding of α-synuclein *in vitro* (237), smaller aggregates *in vivo* (238, 239)	↑mHTT clearance (184) ↑Ser13/Ser16 mHTT phosphorylation (240) ↓cell death (226) ↓ motor, cognitive and psychiatric-like symptoms (241) neurotransmitter level normalization (241)

mHTT, mutated huntingtin.

In all three neurodegenerative diseases, decreases in major gangliosides (GM1, GD1a, GD1b) and increases in minor gangliosides (GM3, GD3) have been reported (Table 3) (189, 190, 191). Although these major brain gangliosides are expressed by all neurons, the neurons primarily affected in each neurodegenerative disease are different: cholinergic neurons in AD (192), dopaminergic neurons in PD, (193), and GABAergic neurons in HD (194). This suggests that changes in ganglioside expression do not initiate disease, but instead modulate disease progress. In contrast, some minor gangliosides are neuron type specific. Cholinergic neurons express GQ1bα and GT1aα, initially called “Chol-1 gangliosides”, whose expression is disrupted in AD and mouse models of AD (195, 196).

Given these findings, gangliosides and ganglioside mimetics are under study as therapies for neurodegenerative diseases (197, 198, 199). Disease-modifying activity of gangliosides has been reported in mouse disease models and, to a limited extent, in human trials (Table 3). Further clinical studies will clarify the potential of gangliosides in the therapy of neurodegenerative diseases (200).

#### Major roles of gangliosides in other human diseases

Gangliosides are directly responsible for two other classes of human diseases, lysosomal storage diseases (201) and autoimmune diseases (202). The first ganglioside isolated and characterized was GM2, which Ernst Klenk extracted from *postmortem* brain tissue of a young patient who died from severe progressive neurodegeneration with intraneuronal lysosomal inclusions (44). The subject suffered from Tay-Sachs disease, a form of GM2 gangliosidosis in which a key enzyme in ganglioside catabolism, hexosaminidase, was defective resulting in extraordinarily high accumulation of the upstream ganglioside, GM2. Several forms of congenital GM2 gangliosidoses and GM1 gangliosidoses have been identified, which vary in onset and severity depending on the mutations responsible. More recently, gangliosides were found to be the target for nervous-system disruption *via* autoimmune disease. Remarkably, some strains of the common diarrheal pathogen *Campylobacter jejuni* carry replicas of the glycan termini of human major gangliosides on their lipo-oligosaccharides. Subjects infected with these strains suffer acute diarrhea, which is cleared immunologically, followed by flaccid paralysis when anti-ganglioside antibodies attack their peripheral nerves. These diseases, which share properties with other lysosomal storage and autoimmune disorders, are not explored further here. Interested readers are referred to excellent reviews (201, 202).

### Concluding statement

As sialoglycans expressed by all vertebrate cells, and the predominant sialoglycans of the mammalian brain, gangliosides are integral to the structure and function of cell surfaces and select intracellular organelles. The diverse severe outcomes in subjects lacking complex gangliosides speak to their functions as mediators and modulators of cell and tissue functions. While mechanisms of some of the physiological and pathological roles of gangliosides are well established, many are not. Enhanced tools for investigating ganglioside regulation of biological pathways continue to be developed. Among these are human induced pluripotent stem cell-derived neurons that carry the same mutations as congenital disorders of ganglioside biosynthesis (203), ganglioside mapping by imaging mass spectrometry (204), and click chemistry tools to map the ganglioside-protein interactome (88). As these and other enhanced tools for investigating the mechanisms of ganglioside regulation of biological pathways are developed, understanding their roles and opportunities to intervene therapeutically by targeting or using gangliosides are likely to expand.

## Conflict of interest

The authors declare that they have no conflicts of interest related to the contents of this article.
